# Population genetic structure of 2 mole species (*Mogera imaizumii* and *M. wogura*) in the Japanese Archipelago

**DOI:** 10.1093/jmammal/gyae157

**Published:** 2025-01-20

**Authors:** Takeru Tsunoi, Gohta Kinoshita, Reiko Mitsuhashi, Masashi Harada, Jun J Sato, Shoji Tatsumoto, Yasuhiro Go, Hitoshi Suzuki, Naoki Osada

**Affiliations:** Graduate School of Information Science and Technology, Hokkaido University, Kita 14, Nishi 9, Kita-ku, Sapporo 060-0814, Japan; Faculty of Science, University of Toyama, Gofuku 3190, Toyama 930-8555, Japan; Graduate School of Environmental Science, Hokkaido University, Kita 10, Nishi 5, Kita-ku, Sapporo 060-0810, Japan; Oji 2-5-17, Kitakatsuragi-gun, Nara 636-0022, Japan; Department of Biological Science, Fukuyama University, Higashimura-cho, Aza, Sanzo, 985, Fukuyama 729-0292, Japan; Cognitive Genomics Research Group, Exploratory Research Center on Life and Living Systems, National Institutes of Natural Sciences, Nishigonaka 38, Myodaiji, Okazaki 444-8585, Japan; Cognitive Genomics Research Group, Exploratory Research Center on Life and Living Systems, National Institutes of Natural Sciences, Nishigonaka 38, Myodaiji, Okazaki 444-8585, Japan; Department of Physiological Sciences, National Institute for Physiological Sciences, Nishigonaka 38, Myodaiji, Okazaki 444-8585, Japan; Graduate School of Information Science, University of Hyogo, 7-1-28 Minatojima-minamimachi, Chuo-ku, Kobe 650-0047, Japan; Graduate School of Environmental Science, Hokkaido University, Kita 10, Nishi 5, Kita-ku, Sapporo 060-0810, Japan; Graduate School of Information Science and Technology, Hokkaido University, Kita 14, Nishi 9, Kita-ku, Sapporo 060-0814, Japan

**Keywords:** Japanese Archipelago, Large Japanese Mole, Lesser Japanese Mole, MIG-seq, *Mogera imaizumii*, *Mogera wogura*, phylogeography, Quaternary, reduced genome representation, whole genome sequencing

## Abstract

The Japanese Archipelago hosts a diverse group of mammalian species, including subterranean moles, whose migratory and dispersion patterns are believed to have been significantly influenced by environmental fluctuations during the Quaternary period. However, the genetic structure of these species has not been extensively studied using large-scale molecular data. In this study, we explored the population structure of 2 widely distributed mole species in Japan: the Lesser Japanese Mole (*Mogera imaizumii*), found mainly in eastern Honshu with a few isolated patches in western Honshu, and the Large Japanese Mole (*M. wogura*), distributed in western Honshu, Shikoku, and Kyushu. We constructed a genome sequence for *M. wogura* using the Chromium system and conducted a reduced genome representation analysis using multiplexed inter-simple sequence repeat genotyping by sequencing on *M. imaizumii* and *M. wogura*. Our findings indicate that *M. imaizumii* comprises 3 major genetic clusters (nc*Mim*- 1 to 3) that are strongly differentiated (fixation index ranging from 0.376 to 0.478). We discovered genetic connections between populations in the southern Kinki area and isolated populations in western Japan. *Mogera wogura* consists of 5 main genetic clusters (nc*Mwo*- 1 to 5). Evidence of introgression between 2 genetic clusters (nc*Mwo*-2/nc*Mwo*-3) was found among individuals from the Chugoku area, indicating secondary contact between the 2 differentiated populations. Both species shared a similar population boundary within the Kinki area, which may be associated with current and past geographic barriers. We confirmed that the Kinki region serves as an important site for the diversification of moles, where multiple factors (topographic barriers, interspecific interactions, and/or isolation related to vegetation) may have shaped their population genetic structures.

## Teaser

Reduced genome representation analysis was conducted on 2 mole species in Japan to determine their population genetic structures.

Understanding the factors that shape current biodiversity patterns is vital for evolutionary, ecological, and conservation biology. Historical climatic fluctuations frequently cause population expansion or contraction, often leading to shifts in species distribution, with glacial cycles being one of the most significant driving forces (Haffer 1969; Bennett and Provan 2008). Genetic diversity and population structure have been extensively studied in a wide range of mammals (Stewart and Cooper 2008) but are largely unexplored in strictly subterranean moles. Although strictly subterranean animals inhabit an environment that is largely insulated from short-term and seasonal climatic fluctuations (Feuda et al. 2015), it is plausible to consider them susceptible to changes in vegetation because the availability of food resources and plant cover in habitats are key factors that influence population dynamics (Nevo 1979). Therefore, vegetation changes caused by past climatic changes may have affected the population dynamics of a subterranean species. Such predictions in subterranean animals have been partly examined by studies of partial mitochondrial genome sequences but have rarely been tested using a genome-wide dataset.

The Japanese Archipelago is home to 8 species of subterranean moles belonging to 4 genera within the family Talpidae (Ohdachi et al. 2009). They inhabit a wide variety of environments, including plains, mountainous forests, riversides, and agricultural lands such as wet and dry crop fields (Abe 1967, 1968; Ohdachi et al. 2009). They are strictly subterranean, burrowing underground and feeding mainly on earthworms and other invertebrates (Imaizumi and Obara 1966; Abe 1968; Yokohata 2005).

The complex topographic features of the archipelago and Pleistocene climatic oscillations played an important role in the history of migration and dispersion in mammals (Sato 2017), with several biogeographic borders being shared across terrestrial species. For example, an important border divides the archipelago into eastern and western parts, as observed in the different karyotypes of large Japanese wood mice (Tsuchiya 1974) and greater Japanese shrew moles (Harada et al. 2004). This border might also have served as the interspecific boundary between the 2 most widespread and abundant species of moles, the Lesser Japanese Mole (*Mogera imaizumii*) and the Large Japanese Mole (*M. wogura*), distributed in the eastern and western parts of Japan, respectively. However, the effects of topographic and climatic variations on the evolutionary processes of subterranean species in Japan remain unclear because of insufficient population genetic analyses.

**Fig. 1.**
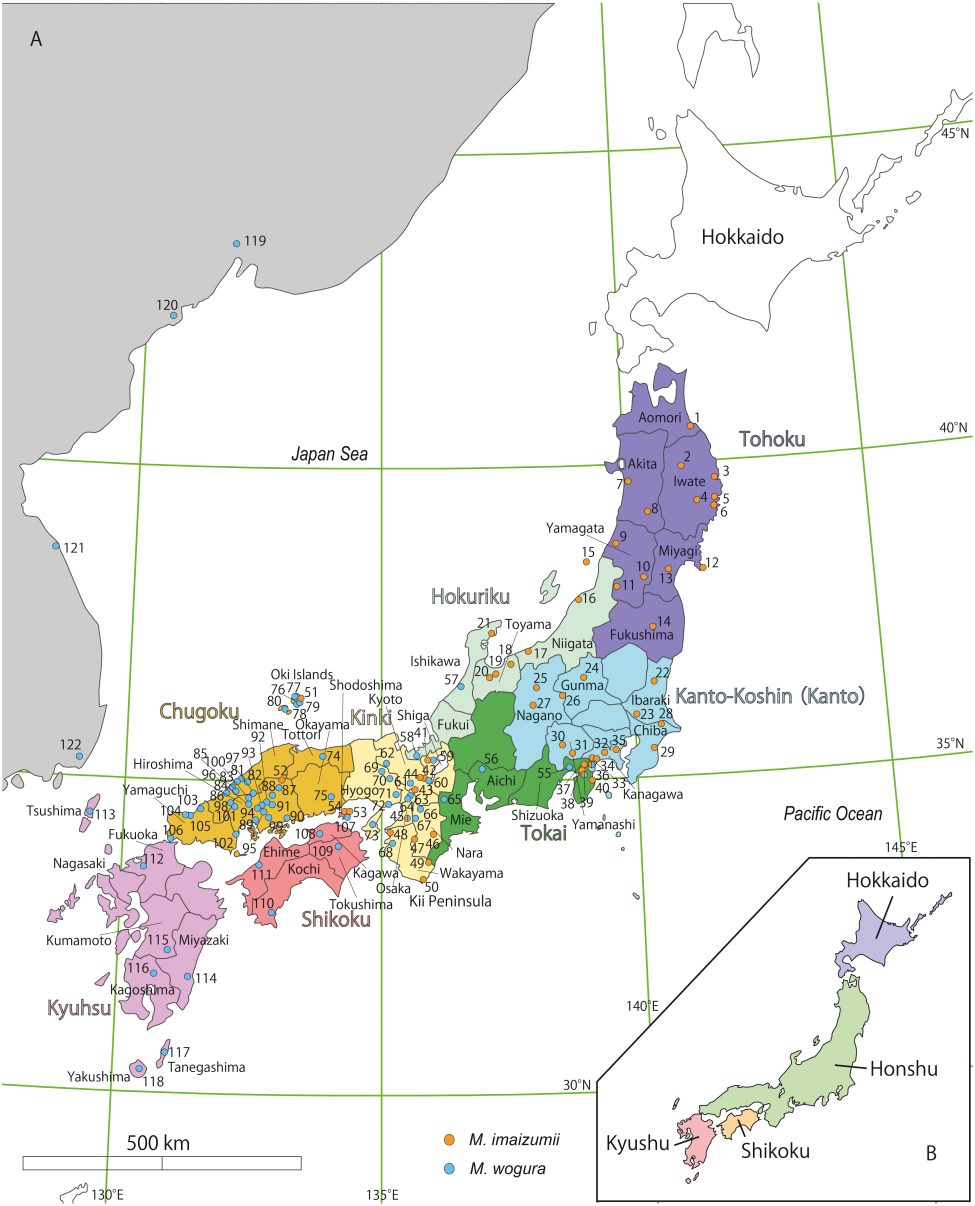
Sampling sites for *Mogera imaizumii* and *M. wogura* A). The map is colored based on geographic districts of Japan (Hokkaido to Kyushu). The borders represent prefectures. The names of 4 main islands in Japan are shown in B). The numbers represent locality numbers (Appendices I and II).

*Mogera imaizumii* and *M. wogura* have been present in the Japanese Archipelago for approximately 2.4 and 1.3 (or 1.6) million years, respectively (Kirihara et al. 2013; Sato 2017). These timeframes include periods of significant environmental change, such as fluctuations in temperature and sea level in response to the 100,000-yr glacial-interglacial cycles during the past approximately 800,000 yr of the Quaternary period. Combined with their wide range of habitats, these 2 species are ideal representatives for analyzing the effects of various topographic and climatic variations on the population structure of subterranean species in Japan. Previous studies based on mitochondrial sequences and limited nuclear gene data have suggested that both species experienced severe population bottlenecks during the Last Glacial Maximum (LGM) and Penultimate Glacial Maximum, 2 crucial epochs marked by drastic cold temperatures (Nakamoto et al. 2021). Based on mitochondrial and nuclear gene sequences, Kirihara et al. (2013) proposed that temperature warming during the Quaternary period prompted the mole distribution border to move eastward. This shift was considered to have left several isolated populations of *M. imaizumii* confined to several small areas in western Japan such as the mountainous areas of the Kinki (including the Kii Peninsula), Chugoku, and Shikoku areas; Shodoshima Island; and small islands such as Oki-Dogo Island (Abe 2001; Tsunoi et al. 2023; Fig. 1). However, this hypothesis has not been tested using large-scale molecular datasets. Therefore, it is unclear whether the eastward movement of *M. imaizumii* and the replacement of *M. imaizumii* by *M. wogura* left a patchy distribution of *M. imaizumii* in western Japan.

Previous studies have shown that, in Japan, both *M. imaizumii* and *M. wogura* consist of several genetically distinct subpopulations (Okamoto 1997; Tsuchiya et al. 2000). Iwasa et al. (2006) identified 2 lineages of *M. imaizumii* in eastern Honshu, each predominantly distributed on the western and eastern coasts respectively, with evidence of some gene flow and exchange between these locations. The lineages are thought to be segregated primarily by high mountain ranges, notably the Ou and Kitakami ranges. Phylogeographic analyses of *M. imaizumii* and *M. wogura* across Japan have been refined using mitochondrial DNA (mtDNA) and nuclear gene sequences from a wide range of samples (Okamoto 1997; Tsuchiya et al. 2000; Kirihara et al. 2013; Zemlemerova et al. 2019; Nakamoto et al. 2021). Phylogenetic analyses of the mitochondrial cytochrome *b* (*Cytb*) gene sequences revealed that *M. imaizumii* and *M. wogura* are both composed of 4 geographically distinct mitochondrial lineages. The 4 mitochondrial lineages of *M. imaizumii*, identified as mt*Mim*-I to IV, were found in distinct geographical areas: the Japan Sea side (Tohoku/Hokuriku areas); Pacific Ocean side (Kanto/Tohoku areas); Kinki area, and an isolated island in western Japan known as Oki-Dogo (Tsunoi et al. 2023; Supplementary Data SD1). Four specific mitochondrial lineages have been identified for *M. wogura*: mt*Mwo*-I in the Tokai and southern Kinki (sKinki) areas; mt*Mwo*-II in the Hokuriku, eastern Chugoku (eChugoku), northern Kinki (nKinki) and Shikoku areas; mt*Mwo*-III in western Chugoku (wChugoku) and Kyushu areas; and mt*Mwo*-IV—which is sometimes identified as a separate species (*M. robusta*)—in coastal regions of the Eurasian continent including Russia and Korea (Nakamoto et al. 2021; Fig. 1). Nakamoto et al. (2021) examined the borders between mitochondrial lineages and identified a geographical boundary between mt*Mwo*-I and mt*Mwo*-II on the Osaka plain and in the Rokko and Hokusetsu mountains. However, no physical barrier was found between mt*Mwo*-II and mt*Mwo*-III, leaving the cause of genetic differentiation between these lineages unexplained. However, elucidating a fine-scale pattern of population differentiation was difficult in prior research because the precise population structures were not clear based only on phenotypic data (e.g., morphology) and a limited amount of molecular data.

Recent advances in next-generation sequencing technology have enabled the acquisition of vast amounts of data from nonmodel organisms, surpassing the traditional reliance on classical genetic markers such as mtDNA, nuclear gene-coding regions, and microsatellites (e.g., Kirihara et al. 2013; Nakamoto et al. 2021). In particular, reduced genome representation methods, such as double-digest restriction site-associated DNA sequencing (ddRAD-seq; Peterson et al. 2012), genotyping by random amplicon sequencing-direct (GRAS-Di; Hosoya et al. 2019), and multiplexed inter-simple sequence repeat genotyping by sequencing (MIG-seq; Suyama and Matsuki 2015), are increasingly being used for genetic studies on mammals in Japan (Ito et al. 2021; Sato and Yasuda 2022; Kinoshita et al. 2024). MIG-seq stands out among these methods because of its cost-effectiveness and capacity to handle a large number of samples, thereby enabling the acquisition of a substantial number of single nucleotide polymorphisms (SNPs), which helps to address the limitations of the variation used in previous genetic analyses. In addition, using information from a reference genome sequence can increase the number of SNPs in the MIG-seq data (Shafer et al. 2016; Kunvar et al. 2021). Currently, no reference genome sequences are available for *M. wogura* or *M. imaizumii*. Therefore, in the present study, we constructed a reference genome sequence for *M. wogura* to enhance the number of SNPs for MIG-seq analysis. Using this newly assembled genome sequence, this study investigated the phylogeographic structure and history of *M. imaizumii* and *M. wogura*, focusing on: (i) analyzing the discordance between mitochondrial and nuclear phylogeographic patterns; (ii) examining the role of geographic barriers in gene flow and introgression among populations; and (iii) identifying traces of recent shifts in species boundaries between *M. imaizumii* and *M. wogura*.

## Methods

### Whole genome sequencing and assembling the draft genome.

An *M. wogura* sample collected in Nara, Japan, was used for whole genome sequencing and assembly. The samples were preserved at −80 °C. The brain, liver, lung, and muscle tissues were used for high-molecular weight (HMW) DNA extraction. HMW DNA was extracted using the Nanobind Tissue Big DNA Kit (Circulomics Inc. #900–001-01) following the manufacturer’s protocol, and fragmented DNA was removed using the Short Read Eliminator (SRE) XL Kit (Circulomics Inc. #SS-100-111-01). The supernatant from the first wash was also centrifuged and washed twice (double SRE; DSRE), followed by elution with 20 µL elution buffer. Ten samples of the extracted HMW DNA with no SRE, SRE, and washed supernatants of SRE were subjected to pulse-field gel electrophoresis (PFGE) using a CHEF-MAPPER system (Bio-Rad). Based on the PFGE band patterns and the DNA integrity number value of the TapeStation (Agilent Technologies), the highest HMW DNA derived from the brain sample was selected for further analysis. A linked-read library was constructed from 1.25 ng of >50 kb HMW DNA using the 10× genomics chromium system and Chromium Genome Reagent Kits v2 (10× Genomics) following the manufacturer’s protocol. The final constructed library was quantified using a TapeStation Bioanalyzer (Agilent Technologies) and Qubit Fluorometer (Thermo Fisher Scientific), and sequenced on an Illumina HiSeq X platform (Illumina, San Diego, USA) with 150 paired-end reads. Sequencing reads were assembled using Supernova version 2.1.1 (Weisenfeld et al. 2017) with default settings.

### MIG-seq library preparation and SNP data generation.

DNA was extracted from tissue samples (liver, muscle, or bone) preserved in 99.8% ethanol using a QIAamp DNA Mini Kit (QIAGEN, Hilden, Germany). DNA extracted from 178 samples (*M. imaizumii*, *n* = 89; *M. wogura*, *n* = 89) collected between 1995 and 2022 were used for MIG-seq analyses. The samples used in this study are shown in Appendices I and II. For the collection of new specimens, we followed ASM guidelines (Sikes et al. 2016). The DNA samples are deposited at the Botanic Garden & Museum, Field Science Center for Northern Biosphere, Hokkaido University, Sapporo (HUNHM; Appendices I and II). Sampling sites covered the main habitats of *M. imaizumii* and *M. wogura*, including the coastal areas of the Asian Continent, where *M. wogura* is sometimes referred to as *M. robusta*. Library construction for MIG-seq was performed following the protocol of Suyama and Matsuki (2015) but with the annealing temperature of the first PCR set to 38 °C. PCR products were run using the MultiNA system (Shimadzu Corporation, Kyoto, Japan) after the first and second PCR to determine whether the DNA was successively amplified. The second PCR products were then pooled and fragments of 300–800 bp were selected using SPRIselect (Beckman Coulter). The constructed library was sequenced with 76 bp paired-end reads using the MiSeq system (Illumina) with the MiSeq Reagent Kit v3 (150 cycles) and 2% PhiX spike-in.

Raw data obtained from the MiSeq system were trimmed using Trimmomatic version 0.39 (Bolger et al. 2014). The first 5 bp and last 1 bp of the reads were trimmed from the raw data using the following parameters: SLIDINGWINDOW, 4:15; CROP, 75; HEADCROP, 5; and MINLEN, 70. The trimmed reads were then mapped onto the *M. wogura* reference genome using the Burrows–Wheeler aligner-maximum exact matches (BWA-MEM) program version 0.7.17 (Li 2013), converted to BAM format by the SAMtools program version 1.7 (Li et al. 2009), and assigned to read-groups using the AddOrReplaceReadGroups function of Picard (Broad Institute, https://broadinstitute.github.io/picard/). Contig generation and SNP detection were performed using *gstacks* analysis implemented in Stacks 2.62 (Catchen et al. 2011, 2013). Contigs that were not shared by 80% of each population and alleles with allele frequencies < 0.01 or heterozygosity > 0.5, were filtered out by *populations*, also implemented in Stacks. Upon conducting *populations*, individuals were assigned to each population based on their sampling sites and mtDNA lineages defined in Nakamoto et al. (2021). Subsequent filtering of samples and SNP sites was performed using TASSEL version 5.2.79 (Bradbury et al. 2007). This process filtered out samples that retained < 80% and < 70% of the SNPs for *M. wogura* and *M. imaizumii,* respectively, as well as SNP sites where minor alleles were observed in only 1 sample. The final all-SNP dataset included 86 samples and 2,988 variable sites for *M. imaizumii*, and 86 samples and 3,550 variable sites for *M. wogura*. Different SNP datasets were simultaneously created for both species by randomly extracting a single SNP per locus and adding a --write-random-snp flag by *populations*. Filtering of samples and SNP sites was also conducted for these datasets under the same conditions, resulting in 86 samples and 1,005 variable sites for *M. imaizumii*, and 86 samples and 998 variable sites for *M. wogura* (single SNP per locus dataset). These single SNP per locus datasets were created for analysis on population structure, since fastSTRUCTURE does not consider linkage disequilibrium between genetic markers (Raj et al. 2014). Conversion of SNP datasets in VCF format into STRUCTURE, FASTA, PHYLIP, and NEXUS formats was conducted using PGDSpider 2.1.1.5 (Lischer and Excoffier 2012).

### Analyses of population structure.

Population assignment analysis was performed using fastSTRUCTURE (Raj et al. 2014) with *K* values (number of clusters) of 2–10 over 20 independent runs for each *K* value using the single SNP per locus dataset. The most likely number of clusters was evaluated using the *chooseK.py* function in fastSTRUCTURE. Principal component analysis (PCA) was performed using the PLINK program 1.90b6.21 version (Shaun Purcell and Christopher Chang; www.cog-genomics.org/plink/1.9/). PCA results were visualized using the R program 4.1.3 version. Phylogenetic relationships within species were visualized using Neighbor-Net analysis implemented in SplitsTree4 4.18.2 version (Huson and Bryant 2006). PCA and Neighbor-Net analyses were performed on the all-SNP dataset. The interspecies fixation index (*F*_ST_) and intraspecies pairwise *F*_ST_ values were calculated using the SMARTPCA algorithm implemented in the EIGENSOFT package (Patterson et al. 2006; Price et al. 2006). Average heterozygosity was calculated for each species and genetic cluster by outputting all sites to a vcf file using *populations* implemented in Stacks 2.67 version (Catchen et al. 2011, 2013) with --vcf-all option. A Maximum Likelihood (ML) tree was constructed with RAxML-NG 1.2.2 version (Kozlov et al. 2019), and the evolutionary model was selected by ModelTest-NG 0.1.7 version (Flouri et al. 2014; Darriba et al. 2020). Invariant sites were removed using the raxml_ascbias.py script obtained from https://github.com/btmartin721/raxml_ascbias. Under the TVM + G4 model, the ML tree was constructed with 1,000 bootstrap replicates.

## Results

The constructed draft genome assembly was 1.85 Gb in size with 58.48 x raw coverage, and the estimated genome size was 2.09 Gb, comprised of 20,579 scaffolds, with 1.63 K scaffolds longer than 10 Kb. The N50 contig size was 109.18 Kb, and the N50 scaffold size was 41.02 Mb. We identified 11,144 (91.1%) complete single-copy BUSCOs and 183 (1.5%) complete duplicated BUSCOs using BUSCO version 5.3.2 (Manni et al. 2021). By mapping MIG-seq reads to the draft genome, we analyzed MIG-seq data from 87 *M. imaizumii* and 86 *M. wogura* samples. In total, we obtained 2,988 and 3,550 SNPs in *M. imaizumii* and *M. wogura*, respectively. The mean effective per-sample coverage at SNP sites was 6.0× for *M. imaizumii* (ranging from 1.8× to 19.1×) and 6.8× for *M. wogura* (ranging from 2.4× to 17.8×). The average rates of mapped reads were 97.54% for *M. imaizumii* and 97.93% for *M. wogura*, suggesting that the reference genome of *M. wogura*, despite being slightly less efficient, is suitable for analyzing *M. imaizumii*. The 2 species shared only 381 SNPs, and the *F*_ST_ value between *M. imaizumii* and *M. wogura* was 0.868, indicating that they were genetically well-differentiated, although they were sister species. We first constructed a Neighbor-Net graph and confirmed that the 2 species were genetically well separated and differentiated without any sign of hybridization and introgression (Supplementary Data SD2). The ML tree also showed that the 2 species are separated with a high bootstrap score (bootstrap score rate = 100; Supplementary Data SD3). Therefore, we present the results of genetic differentiation among the populations of each species in the subsequent subsections.

### Genetic structure of *Mogera imaizumii.*

**Fig. 2.**
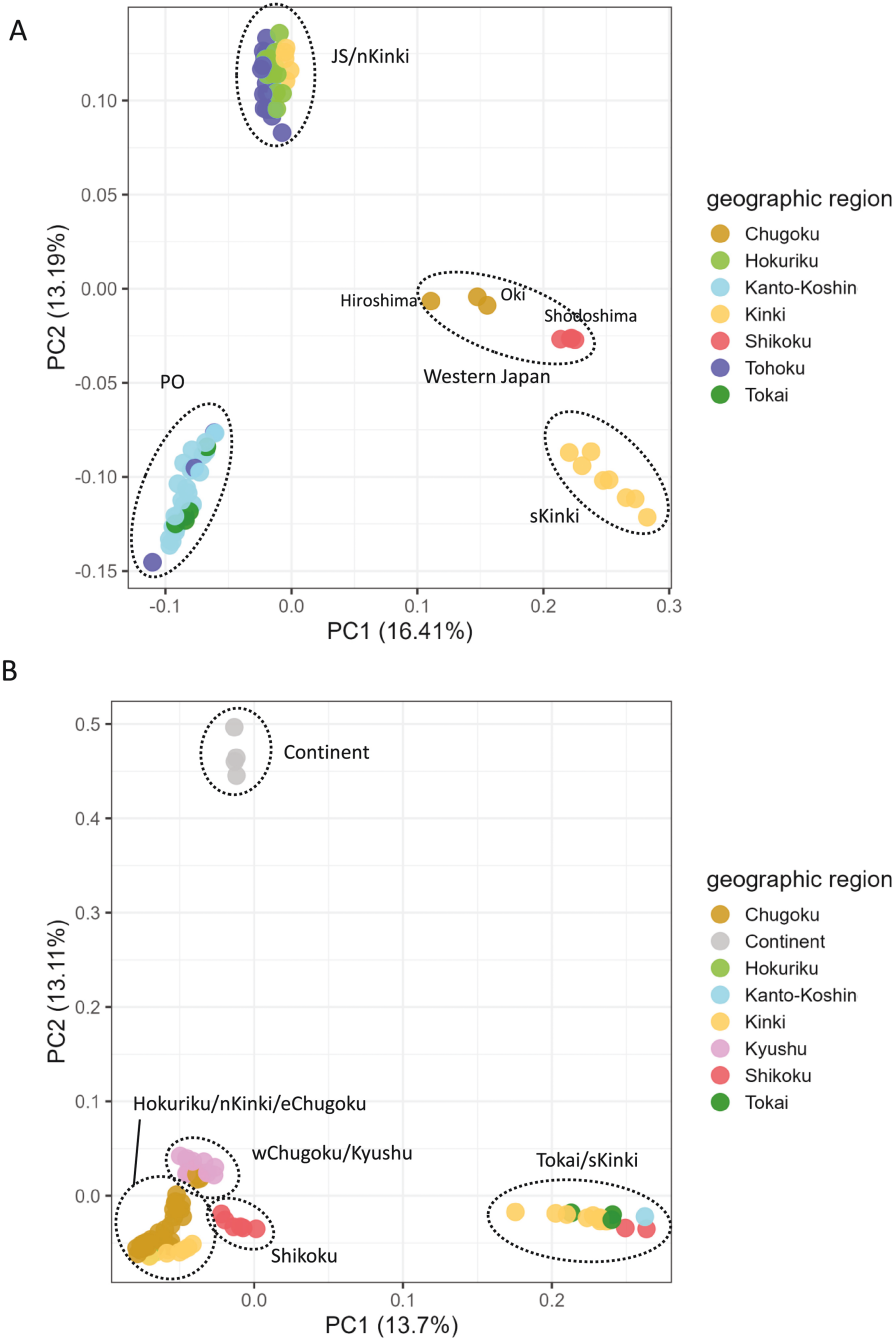
PCA of *Mogera imaizumii* A) and *M. wogura* B) using PLINK. The percentages of axis labels represent eigenvalues of the principal components. Dots are colored according to the geographic districts of their sampling sites. Abbreviations are as follows: JS, Japan Sea side (Aomori, Iwate, Akita, Yamagata, Niigata, Nagano, Toyama, and Ishikawa); PO, Pacific Ocean side (Miyagi, Fukushima, Ibaraki, Chiba, Gunma, Kanagawa, Yamanashi, and Shizuoka); nKinki, northern Kinki (Shiga, Kyoto, and Hyogo); sKinki, southern Kinki (Osaka, Wakayama, and Nara); eChugoku, eastern Chugoku (Tottori, Okayama, Shimane, and Hiroshima); wChugoku, western Chugoku (Yamaguchi). The names of regions and prefectures in Japan are shown in Fig. 1.

**Fig. 3.**
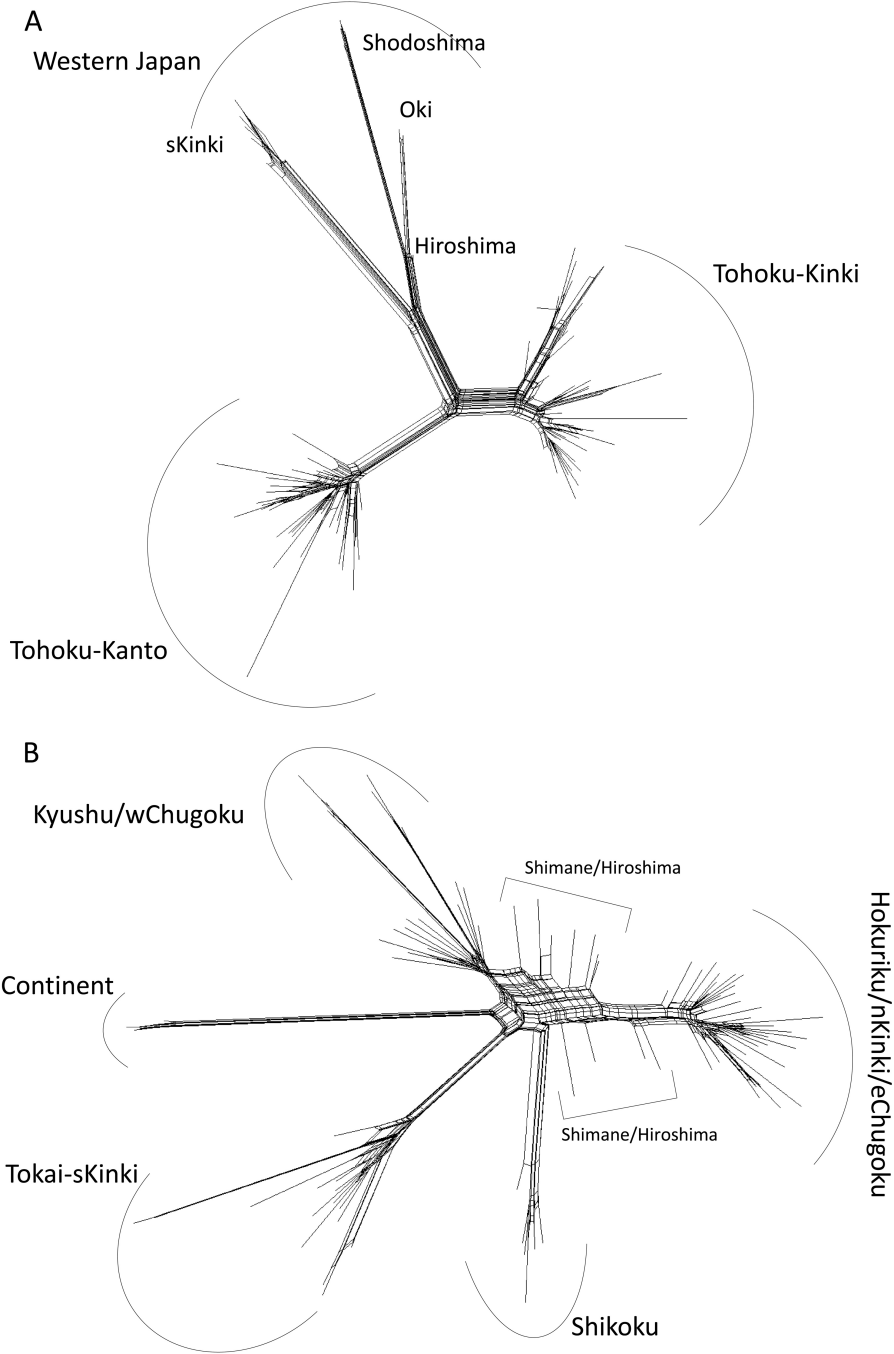
Neighbor-Net analysis constructed by SplitsTree4 for *Mogera imaizumii* A) and *M. wogura* B).

**Fig. 4.**
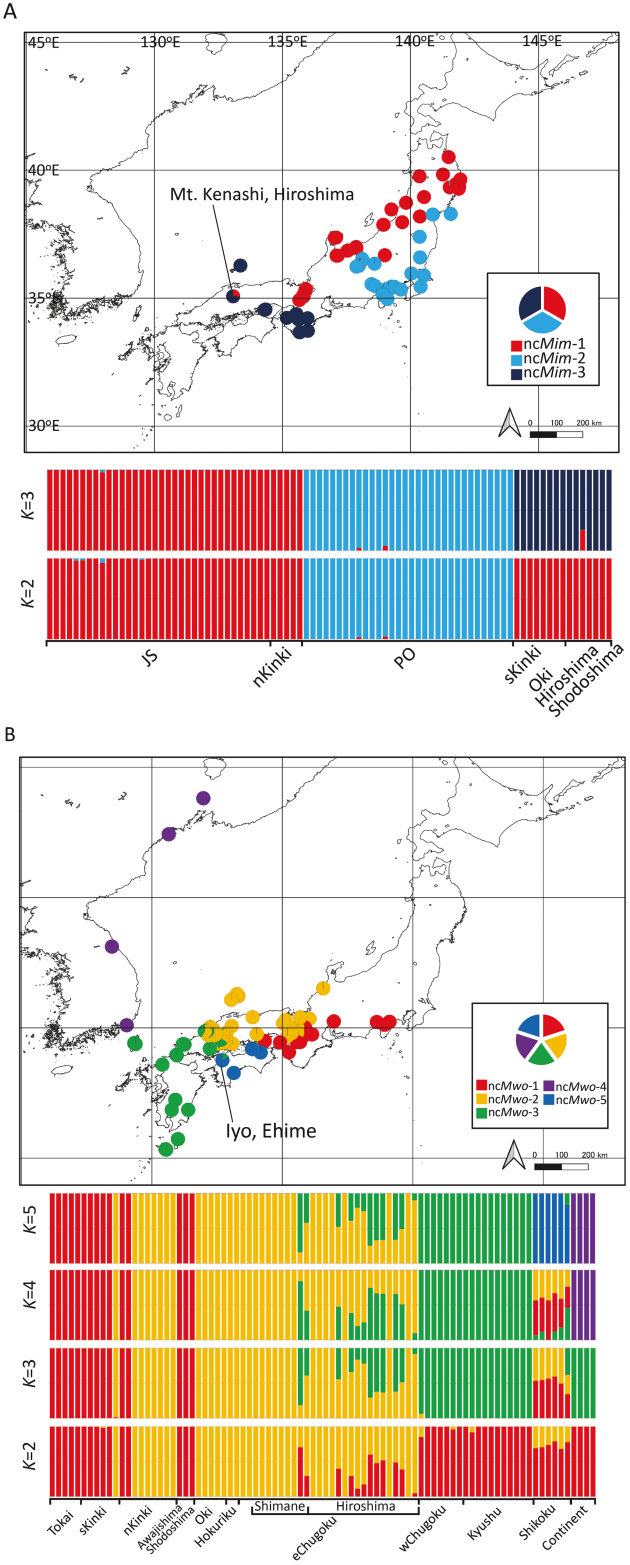
Proportion of admixture determined by fastSTRUCTURE for *Mogera imaizumii* A) and *M. wogura* B). Each pie chart or bar represents an individual, and the coordinates on the map show sampling sites. The coastline data were obtained from the GSHHG at https://www.ngdc.noaa.gov/mgg/shorelines/.

PCA based on the SNPs of *M. imaizumii* showed that PC1 and PC2 segregated the samples into approximately 4 to 6 groups (Fig. 2a). PC1 separated populations from the sKinki area (Osaka, Nara, Wakayama, and Mie) and isolated populations (Shodoshima, Oki, and Hiroshima) from other samples. Individuals from the nKinki area (Shiga, Kyoto, and Hyogo) were grouped with other individuals from the Japan Sea coast (JS; Aomori, Iwate, Akita, Yamagata, Niigata, Nagano, Toyama, and Ishikawa; Fig. 2b) and differentiated from those from the Pacific Ocean coast (PO; Miyagi, Fukushima, Ibaraki, Chiba, Gunma, Kanagawa, Yamanashi, and Shizuoka) on PC2. PC3 and PC4 further separated populations from Oki, Shodoshima, and Hiroshima from the sKinki group (Supplementary Data SD4). PC5 distinguished individuals from JS and nKinki by grouping the Ishikawa population together with the nKinki population, whereas samples from Toyama were interspersed between JS and nKinki (Supplementary Data). The Neighbor-Net graph presented in Fig. 3a is in good agreement with the PCA results, in which the individuals were separated into 3 to 6 major clades. The results of fastSTRUCTURE analyses showed that the value of *K* that maximized the marginal likelihood was 3 for *M. imaizumii*. The populations of JS/nKinki, PO, and sKinki (Hiroshima, Shodoshima, and Oki) were mainly composed of nc*Mim*-1, nc*Mim*-2, and nc*Mim-*3, respectively (Fig. 4a). Although most samples were assigned to a single genetic cluster, an individual from Hiroshima showed signs of admixed ancestry between nc*Mim*-1 and nc*Mim*-3 (Fig. 4a). The sKinki populations were similar to the 3 isolated populations (Oki, Shodoshima, and Hiroshima) in terms of nc*Mim*-3, indicating that the nKinki/sKinki border is also a border between ncMim-1/ncMim-3 genetic groups. Pairwise *F*_ST_ values were high, ranging from 0.376 to 0.487 (Supplementary Data SD5). The average heterozygosity is summarized in Supplementary Data SD7.

### Genetic structure of *Mogera wogura.*

For *M. wogura*, PC1 and PC2 separated populations from the Tokai/sKinki, Hokuriku/nKinki/Chugoku/Shikoku/Kyushu, and continental regions into distinct clusters (Fig. 2b). PC3 reflected genetic variation in individuals from the Chugoku and Kyushu areas (Supplementary Data SD4D); PC4 separated Shikoku individuals from the others (Supplementary Data SD4E); and PC5 separated Tsushima, Yakushima, and Tanegashima Island individuals from the other samples within the Kyushu area (Supplementary Data SD4F). The Neighbor-Net graph shows a complex network separated into 5 major clusters. Several individuals from Shimane and Hiroshima showed signs of hybridization or introgression between Hokuriku/nKinki/eChugoku and Kyushu/wChugoku clusters (Fig. 3b). The fastSTRUCTURE analysis showed that the value of *K* that maximized the marginal likelihood was 5 and identified the 5 most probable genetic clusters as nc*Mwo*-1, nc*Mwo*-2, nc*Mwo*-3, nc*Mwo*-4, and nc*Mwo*-5—mainly observed in the Tokai/sKinki, Hokuriku/nKinki/eChugoku, wChugoku/Kyushu, continental, and Shikoku populations, respectively (Fig. 4b). This result aligns well with the mtDNA haplotype groups defined in a previous study (Nakamoto et al. 2021); however, individuals from the Shikoku region were assigned to the fifth distinct genetic cluster, nc*Mwo*-5 (Fig. 4b). Consistent with the Neighbor-Net results, some individuals from Shimane and Hiroshima showed mixed ancestry of nc*Mwo*-2 and nc*Mwo*-3 (Fig. 4b). These admixed individuals were collected from the distribution border of nc*Mwo*-2 and nc*Mwo*-3, most of which are hosts of the mt*Mwo*-III mtDNA haplotypes (Nakamoto et al.2021). Pairwise *F*_ST_ values were very high between continental and Japanese populations, ranging from 0.597 to 0.701. The lowest *F*_ST_ was observed between nc*Mwo*-2 and nc*Mwo*-3 because these 2 populations showed signs of introgression (Supplementary Data SD6). Average heterozygosity of each genetic cluster is summarized in Supplementary Data SD7. Heterozygosity for nc*Mwo*-4 was considerably low compared to other genetic clusters.

## Discussion

### Population structure of *Mogera imaizumii.*

From the PCA, Neighbor-Net, and fastSTRUCTURE results, *M. imaizumii* was consistently divided into 3 genetically distinguishable clusters. Pairwise *F*_ST_ values also showed that the genetic groups were well-differentiated from each other (Supplementary Data SD5). Iwasa et al. (2006) demonstrated that *M. imaizumii* from eastern Japan comprises 2 genetically distinct groups, as determined by mitochondrial *Cytb* sequences and restriction fragment length polymorphism analysis of the nuclear 28S ribosomal RNA gene spacer. Our results from PCA (Fig. 2) and fastSTRUCTURE (Fig. 4), as well as the high *F*_ST_ values (Supplementary Data SD5), obtained from the MIG-seq analyses of nuclear DNA show high differentiation between *M. imaizumii* groups nc*Mim*-1 and nc*Mim*-2, which also supports the hypothesis that the mountain chain in eastern Japan that runs from north to south through the boundary of nc*Mim*-1 and nc*Mim*-2 distributions acts as a barrier (Iwasa et al. 2006). Iwasa et al. (2006) proposed a secondary contact model, suggesting that these 2 lineages of moles were dispersed via 2 distinct routes (along the coastlines of the JS and PO) and converged in Iwate Prefecture where evidence of gene introgression was observed. However, no strong gene introgression was observed between nc*Mim*-1 and nc*Mim*-2. The isolated populations from Oki, Hiroshima, and Shodoshima were also differentiated from each other, possibly because of genetic drift subsequent to their isolation, as implied by their relatively low heterozygosity (Oki = 0.00050; Hiroshima = 0.00152; Shodoshima = 0.00030). Among the isolated population samples, 1 individual from the Chugoku Mountains (Mt. Kenashi, Hiroshima) exhibited an admixed feature (*K* = 3; Fig. 4a), which may be a trace of the mixed ancestry of this sample with nc*Mim*-1 and nc*Mim*-3 genes. It is noteworthy that the patchy distribution of *M. imaizumii* in Mt. Kenashi, Hiroshima is now entirely encircled by the distribution of *M. wogura*. This pattern implies that *M. imaizumii* populations expanded their distributions westward during the cold periods of the Quaternary because this species, now distributed in the northern parts of Japan, is better adapted to cold environments than is *M. wogura* (Kirihara et al. 2013; Nakamoto et al. 2021). During colder periods, we assumed that the distribution areas of nc*Mim*-1 shifted westward from the Kinki region to the Chugoku region and were mixed with a population related to nc*Mim*-3. Subsequently, as temperatures increased during the postglacial periods and *M. imaizumii* populations began to migrate eastward, nc*Mim*-1 individuals may have remained isolated in their current locations, persisting as admixed populations. However, one cannot ignore that fastSTRUCTURE requires a certain sample size relative to underlying population structure (Raj et al. 2014). Considering that there was only 1 individual available for this *M. wogura* population from Chugoku Mountains, it is possible this mixed ancestry is merely an artifact. Further sampling and analysis of *M. imaizumii* in this isolated area is required to test the proposed hypothesis.

Nakamoto et al. (2021) grouped *M. imaizumii* from the Toyama, Ishikawa, and Kinki areas based on the mtDNA haplotype mt*Mim*-III. Kirihara et al. (2013) suggested that the distribution of *M. imaizumii* shifted in response to climatic changes during the Quaternary and expanded southward during colder glacial periods. While this could explain how the mtDNA haplotype mt*Mim*-III is shared among the Hokuriku/nKinki/sKinki populations, our results based on the nuclear genome indicated that the Hokuriku and nKinki populations clustered with the JS population (main genetic cluster = nc*Mim*-1), whereas the sKinki samples were grouped with the isolated populations (main genetic cluster = nc*Mim*-3). The striking discrepancy between the patterns of mtDNA and nuclear DNA differentiation cannot be readily explained by a simple scenario in which the populations are divided only once and differentiated solely on their own, indicating that there was at least some level of interaction between the diverse populations. This discrepancy can be explained in 2 ways: (i) mitochondrial introgression from the Hokuriku/nKinki population to the sKinki populations; or (ii) 2 routes of nuclear introgression—from the JS population to the Hokuriku/nKinki population and from the isolated population to the sKinki population. These 2 scenarios cannot be distinguished from our data and therefore require further investigation at the genomic scale.

### Population structure of *Mogera wogura.*

From PCA, Neighbor-Net, and fastSTRUCTURE analyses *M. wogura* was categorized into 5 genetic clusters (nc*Mwo*-1 to 5), and a sign of introgression was detected in the Chugoku area. In this study, we identified seemingly genetically admixed individuals at the boundary between these 2 populations, spanning Shimane and Hiroshima prefectures (Supplementary Data SD8). This pattern contrasts with the boundary of the nKinki/sKinki populations, where no clear sign of admixture was observed (Supplementary Data SD8), except that an individual was observed to hold the mt*Mwo*-I haplotype and was mainly assigned to nc*Mwo*-2 genetic cluster (HS4642; Appendix II). It is notable that there seem to be no clear abiotic barriers, such as mountains or rivers, that segregate eChugoku/wChugoku populations. The boundary between these 2 populations was previously identified based on distribution of the mt*Mwo*-II and mt*Mwo*-III mitochondrial haplotypes. However, no signature of introgression was observed in mtDNA, owing to a lack of recombination (Nakamoto et al. 2021). Since most of these genetically admixed individuals contained mtDNA of the mt*Mwo*-III haplotype (Appendix II), this introgression would be unidirectional, either nc*Mwo*-3 to nc*Mwo*-2 or vice versa. Although it remains unclear whether sex-biased dispersal occurs in moles (Satoh et al. 2019), if male-biased gene flow is present, as is the case for most mammals (Greenwood 1980), the observed pattern suggests male-biased gene flow from nc*Mwo*-2- to nc*Mwo*-3-dominated populations. This type of asymmetric introgression is common in secondary contact zones of differentiated populations (Chan and Levin 2005; Currat et al. 2008; Toews and Brelsford 2012; Johnson et al. 2015). Therefore, it is presumed that this boundary is a case in which 2 populations make secondary contact after their isolation. A large river (Gono River) runs east of the boundary (Supplementary Data SD8), and this may have acted as a barrier that did not completely, but significantly, hinder the migration of moles. Nakamoto et al. (2021) showed that *M. wogura* populations in the eChugoku/wChugoku region exhibited signs of recent rapid population expansion, which could have allowed them to cross this barrier, possibly making a detour around the river source and admixing with each other.

Admixture analysis using fastSTRUCTURE revealed that the *M. wogura* population in the Shikoku area constitutes the distinct genetic cluster nc*Mwo*-5. This finding contrasts with those of previous studies based on mtDNA and nuclear gene sequences, which grouped the Shikoku population with Hokuriku, nKinki, and eChugoku populations (Tsuchiya et al. 2000; Kirihara et al. 2013; Nakamoto et al. 2021). The results of this study showed that the Shikoku population has a distinct genetic cluster in terms of nuclear genomes. The high *F*_ST_ values (Supplementary Data SD6) also support that the Shikoku population is well-differentiated, which implies that the Shikoku population may have been isolated from Honshu for a significant amount of time. The common ancestor of the Shikoku population may have immigrated to Shikoku when the Seto Inland Sea (the inland sea between the main islands of Honshu, Shikoku, and Kyushu; Supplementary Data SD8) was absent during the last glacial period (Rohling et al. 1998; Clark and Mix 2002; Oba and Irino 2012; Sato and Yasuda 2022), but further examination is required to estimate their divergence time.

### Common genetic boundary in the Kinki area observed in both *Mogera wogura* and *Mogera imaizumii.*

In this study, we observed a well-defined population boundary in the middle of the Kinki area (from Kobe through northern Osaka to the southernmost tip of Lake Biwa) of *M. wogura* (nc*Mwo*-1*/*nc*Mwo*-2; Supplementary Data SD8), which was also observed in mitochondrial data reported by Nakamoto et al. (2021). This borderline also applies to the boundary between nc*Mim*-1 and nc*Mim*-3 in *M. imaizumii,* as discussed previously. The border is potentially associated with 2 distinct events occurring at different time scales. First, the Jomon transgression, a sea-level rise spanning from the early Pleistocene to the middle Holocene (Umitsu 1991; Ishiga et al. 2000) has been proposed by Nakamoto et al. (2021). Second, the emergence of the second Seto Inland Sea (currently the Osaka plain), an inland water body that formed approximately 3 Ma and persisted until at least the middle Pleistocene (Sugiyama 1991; Yoshida 1992), is also a candidate that could have contributed to the formation of this border.

The dynamic geographic history that the Osaka plain has undergone is very likely to have influenced the distribution of moles. Kirihara et al. (2013) also proposed that the genetic distinctiveness of the *M. wogura* population from Tokai/Kinki (in this case, nc*Mwo*-1) could be the result of the Hokuriku/nKinki *M. imaizumii* population expanding its range southwards, serving as a barrier to prevent *M. wogura* migration. The differences in vegetation in the Kinki region, which is mostly dominated by evergreen broad-leaved forests, include a belt of temperate mixed deciduous forests generally corresponding to the border of the nKinki/sKinki region (Dobson 1994). The vegetation in the belt shifted to a boreal coniferous forest in the LGM and could have acted as a barrier to prevent interactions between *M. wogura* Tokai/Kinki and *M. imaizumii* nKinki/sKinki populations.

We failed to find similar genetic borders in previous studies on other mammalian species, and therefore speculated that this was a unique biogeographic border that could be applied only to moles, at least for now, indicating the presence of an underground-specific factor affecting the dispersion and distribution of subterranean fauna. The weights of these factors cannot be determined based on our data; however, it is possible that these factors act in a complex manner to shape this genetic boundary. Our findings provide new insights into the unique biogeographical features of the Kinki region.

### Taxonomic identity of genetic clusters.

Pairwise *F*_ST_ values between mole populations are considerably high, ranging from 0.376 to 0.487 for *M. imaizumii* (Supplementary Data SD5) and from 0.291 to 0.757 for *M. wogura* (Supplementary Data SD6). This pattern leads us to question the uniformity of each of these moles as a single species. There have been attempts to divide them into separate species based on morphological traits, but they were eventually integrated into the current recognitions of 2 species. Our findings suggest that a reassessment of their classification may be necessary. The same can be said for the continental population of *M. wogura*, whose taxonomic identity is still argued. This issue is expected to be focused on in our future study based on whole genome data.

## Supplementary data

Supplementary data are available at *Journal of Mammalogy* online.

**Supplementary Data SD1.** Geographic distributions of mitochondrial lineages of *M. imaizumii* and *M. wogura* based on previous studies (Nakamoto et al. 2021; Tsunoi et al. 2023; Mitsuhashi et al. in press) and data obtained from IUCN Red List website (https://www.iucnredlist.org/species/41466/22323489).

**Supplementary Data SD2.** Neighbor-Net constructed using SplitsTree4 using *Mogera imaizumii* and *M. wogura* samples.

**Supplementary Data SD3.** Maximum Likelihood tree constructed using RAxML-NG 1.2.2 version (Kozlov et al. 2019). Evolutionary model selection was done by ModelTest-NG 0.1.7 version (Flouri et al. 2014; Darriba et al. 2020). Invariant sites were removed using the raxml_ascbias.py script obtained from https://github.com/btmartin721/raxml_ascbias. Under the TVM + G4 model, ML tree was constructed with 1000 bootstrap replicates. Branch lengths represent the bootstrap score rates.

**Supplementary Data SD4.** PCA plots of PC3-PC5 to PC1 for *Mogera imaizumii* (a–c) and *M. wogura* (d–f). The abbreviations are as follows: JS, Japan Sea side (Aomori, Iwate, Akita, Yamagata, Niigata, Nagano, Toyama, and Ishikawa); PO, Pacific Ocean side (Miyagi, Fukushima, Ibaraki, Chiba, Gunma, Kanagawa, Yamanashi, and Shizuoka); nKinki, northern Kinki (Shiga, Kyoto, and Hyogo); sKinki, southern Kinki (Osaka, Wakayama, and Nara). For the names of the regions and prefectures of Japan, see Fig. 1.

**Supplementary Data SD5.** Pairwise *F*_ST_ values for *Mogera imaizumii*.

**Supplementary Data SD6.** Pairwise *F*_ST_ values for *Mogera wogura*.

**Supplementary Data SD7.** Average heterozygosity for each species and genetic cluster.

**Supplementary Data SD8.** A close-up figure of the nc*Mwo*-1/nc*Mwo*-2 (a) and nc*Mwo*-2/nc*Mwo*-3 (b) population borders with the proportion of admixture determined using fastSTRUCTURE for *Mogera wogura*. The coastline data were obtained from the GSHHG at https://www.ngdc.noaa.gov/mgg/shorelines/. The altitude data were obtained from the National Land Information Division, National Spatial Planning and Regional Policy Bureau, MLIT of Japan at https://nlftp.mlit.go.jp/ksj/gml/datalist/KsjTmplt-G04-a.html. The river data were obtained from the Geospatial Information Authority of Japan at https://www.gsi.go.jp/kankyochiri/gm_japan_e.html. Numbers represent the locality numbers (Appendix II).

gyae157_suppl_Supplementary_Datas_SD1

gyae157_suppl_Supplementary_Datas_SD2

gyae157_suppl_Supplementary_Datas_SD3

gyae157_suppl_Supplementary_Datas_SD4

gyae157_suppl_Supplementary_Datas_SD5

gyae157_suppl_Supplementary_Datas_SD6

gyae157_suppl_Supplementary_Datas_SD7

gyae157_suppl_Supplementary_Datas_SD8

gyae157_suppl_Supplementary_Appendix_1

gyae157_suppl_Supplementary_Appendix_2

## Data Availability

The sequenced 10x Chromium linked-reads were deposited in the DNA Data Bank of Japan Center (DDBJ) and are available under accession numbers BAABMH010000001–BAABMH010020570. Raw FASTQ data from MIG-seq were also deposited in DDBJ and are available under accession numbers PRJDB17819 (DRR545064–DRR545159, DRR545214–DRR545312).

## Appendix I

List of *Mogera imaizumii* samples used in this study.

## Appendix II

List of *Mogera wogura* samples used in this study.
