# Supplementary figures and images for: Population genetic structure of 2 mole species (Mogera imaizumii and M. wogura) in the Japanese Archipelago
Source: J Mammal. 2025 Jan 20;106(3):576–86. doi: 10.1093/jmammal/gyae157 (PMC12159533; doi:10.1093/jmammal/gyae157)

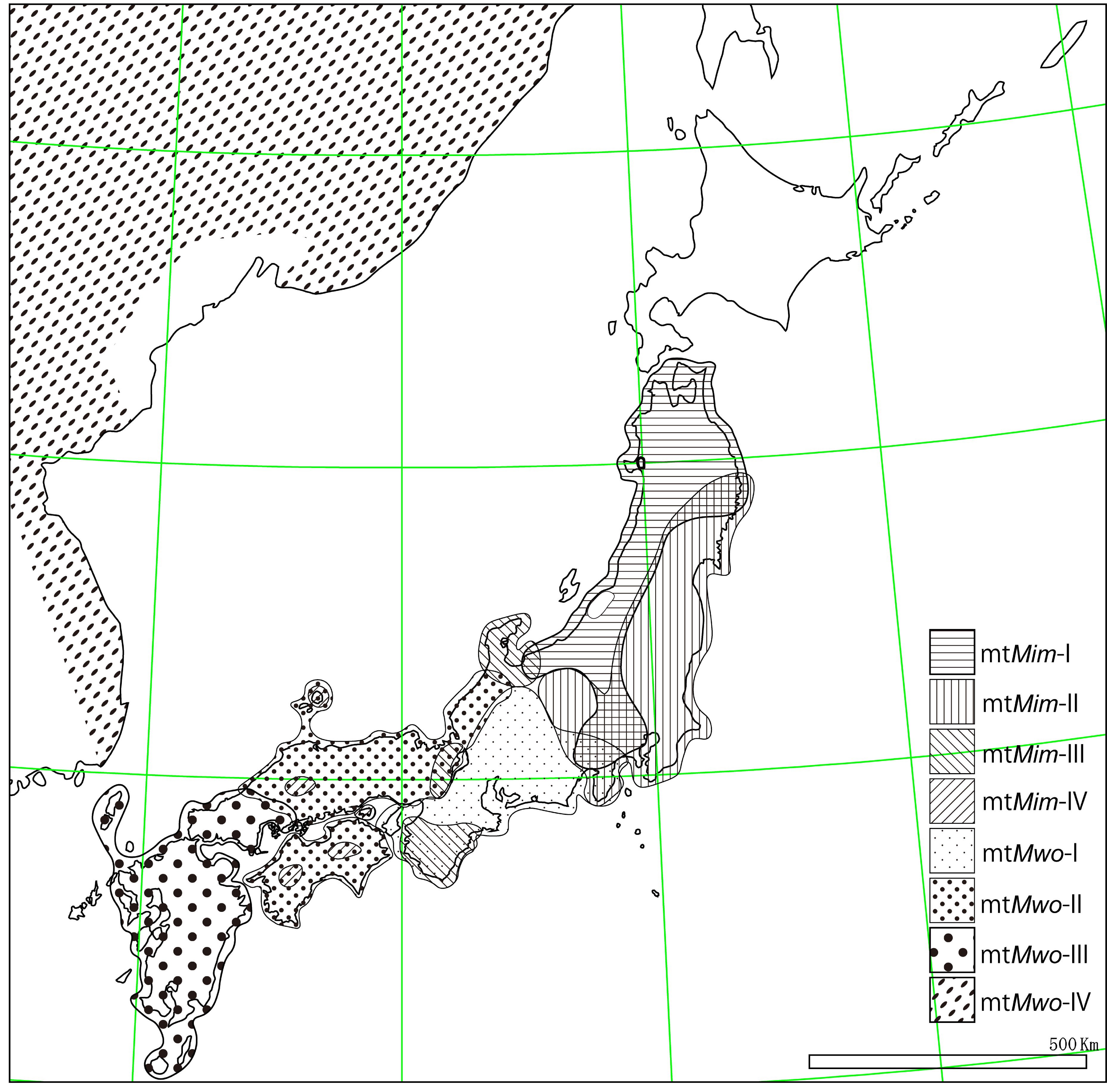

Supplement: gyae157_suppl_Supplementary_Datas_SD1 [file gyae157_suppl_supplementary_datas_sd1.jpeg]

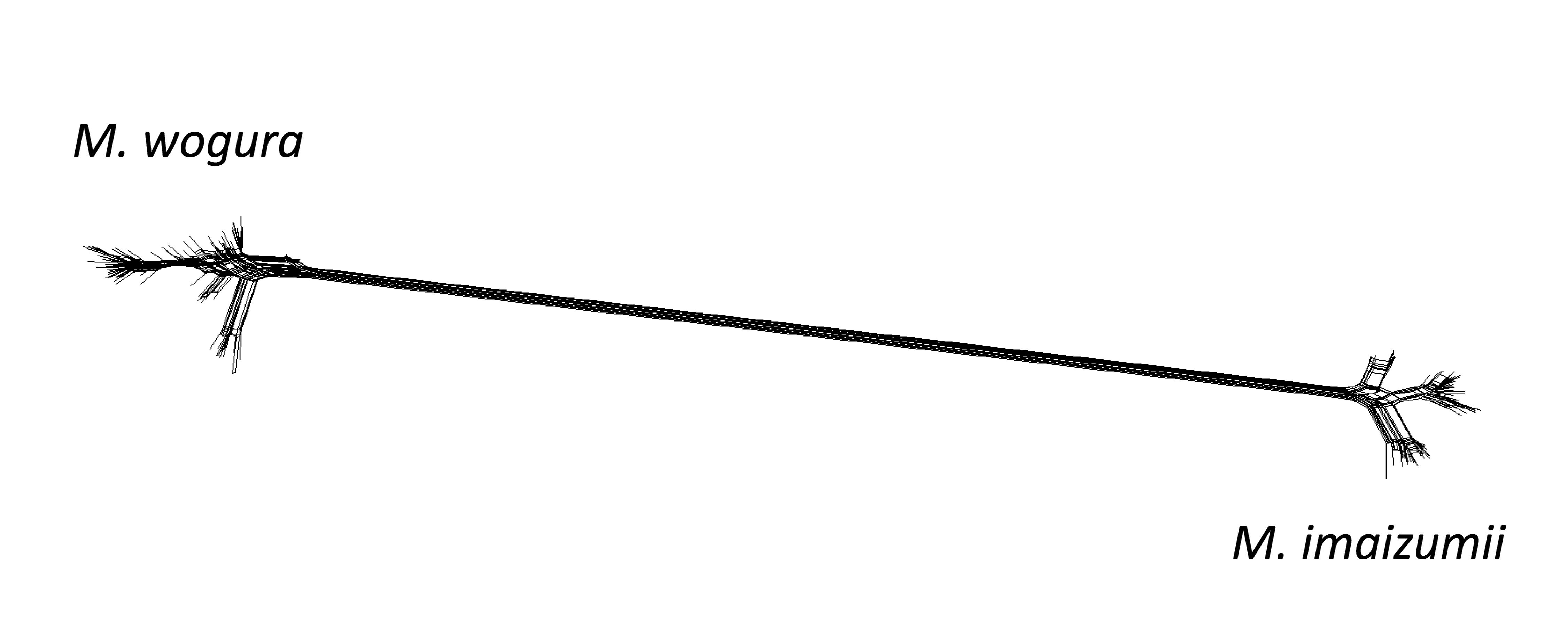

Supplement: gyae157_suppl_Supplementary_Datas_SD2 [file gyae157_suppl_supplementary_datas_sd2.jpeg]

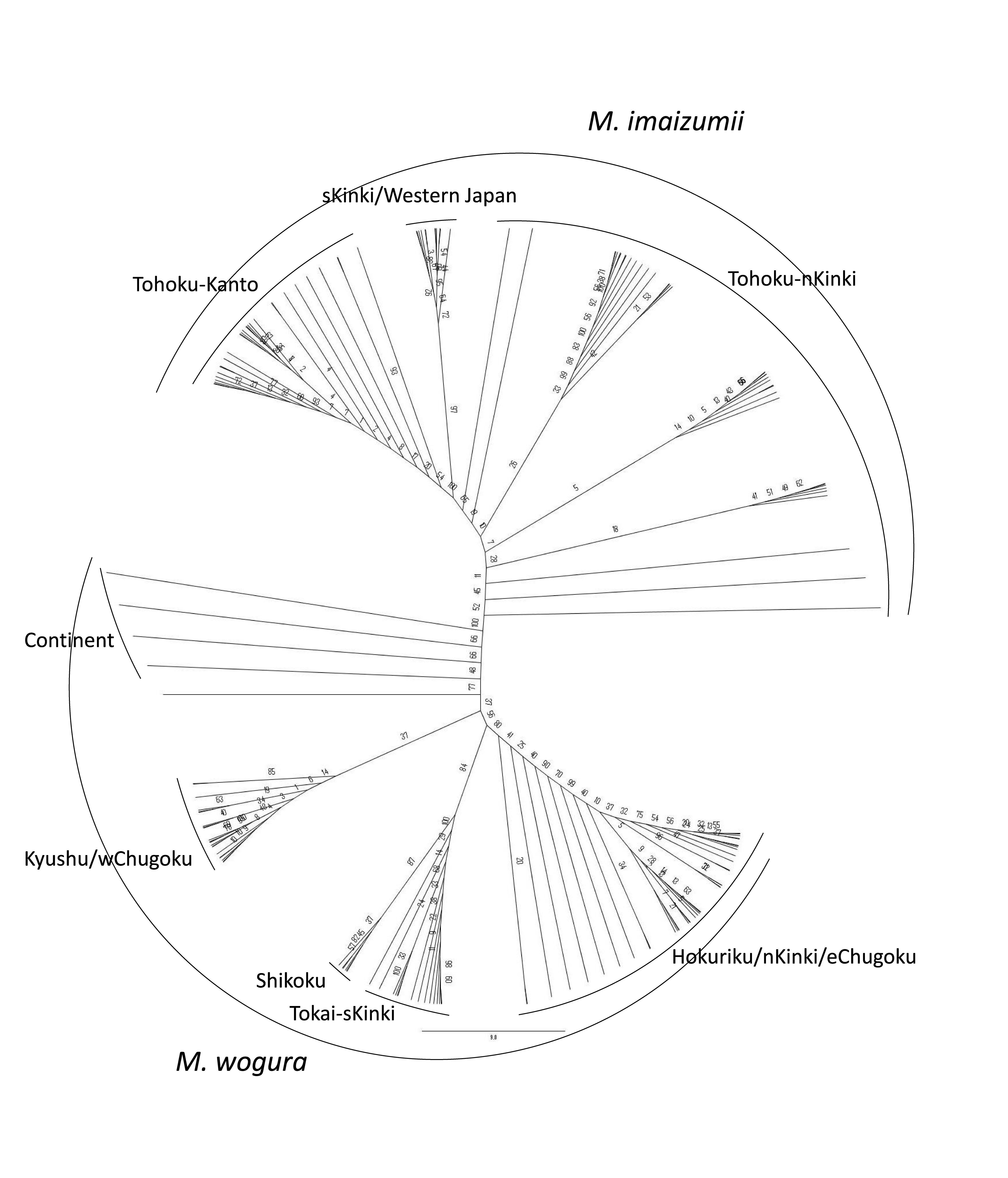

Supplement: gyae157_suppl_Supplementary_Datas_SD3 [file gyae157_suppl_supplementary_datas_sd3.jpeg]

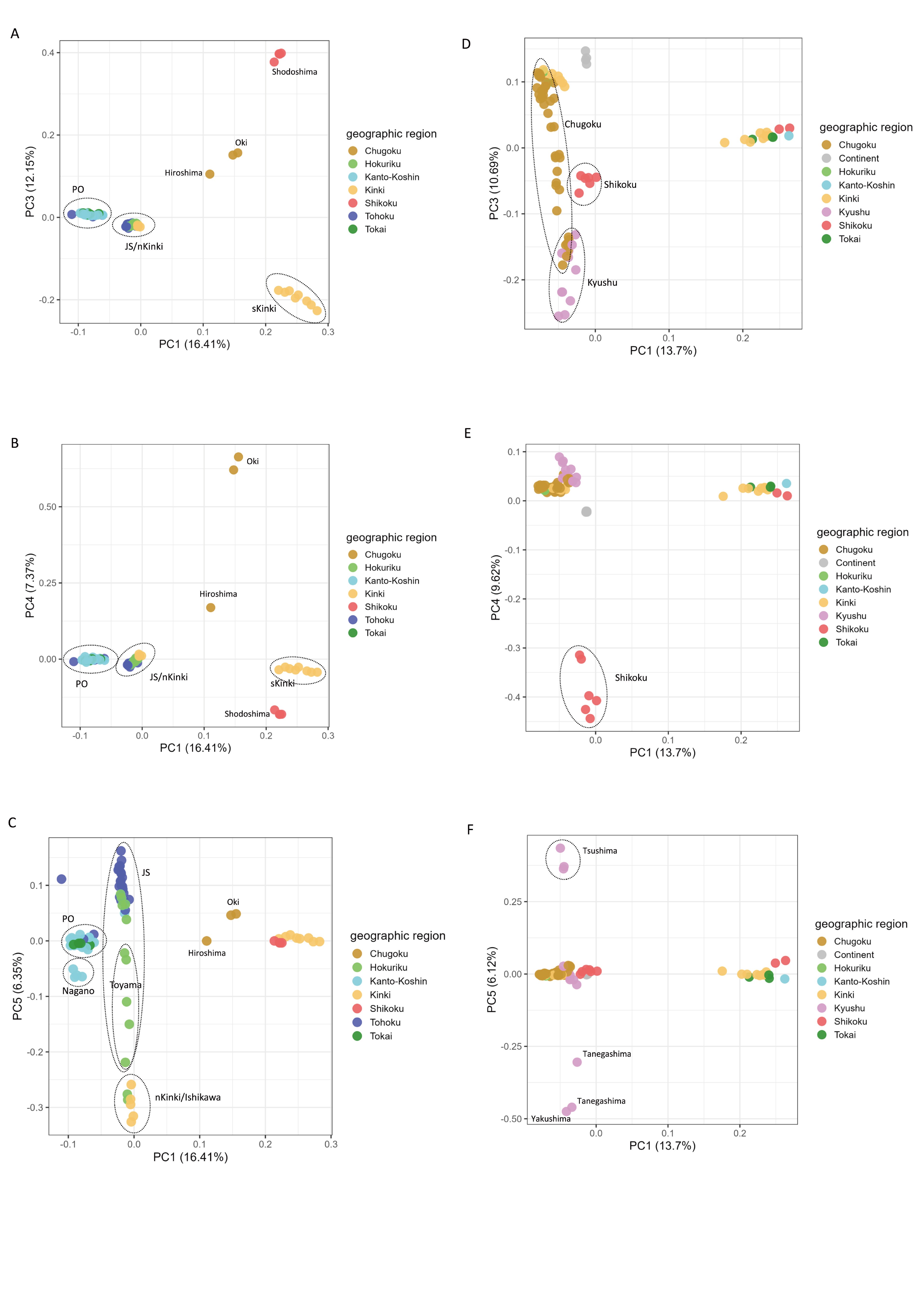

Supplement: gyae157_suppl_Supplementary_Datas_SD4 [file gyae157_suppl_supplementary_datas_sd4.jpeg]

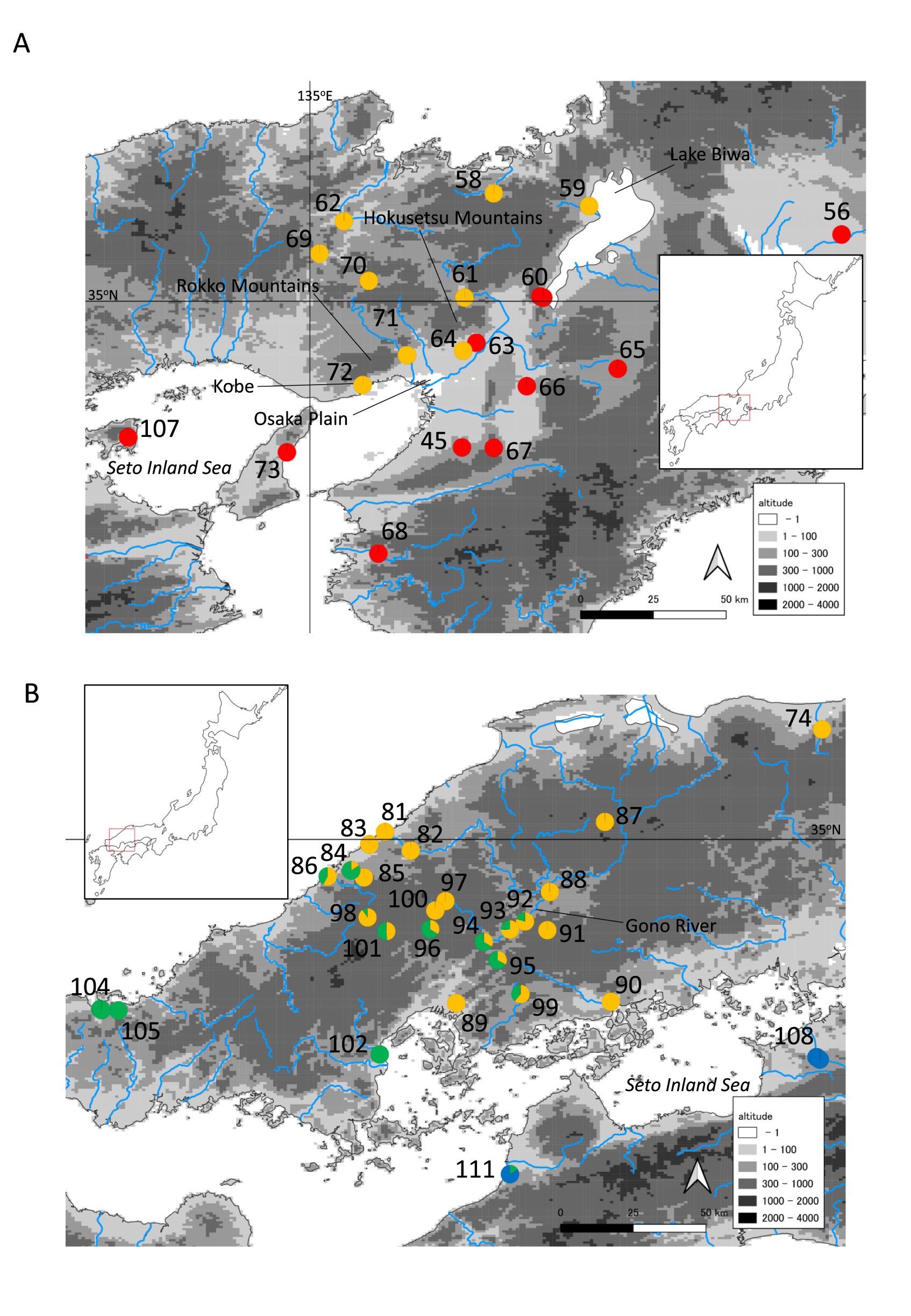

Supplement: gyae157_suppl_Supplementary_Datas_SD8 [file gyae157_suppl_supplementary_datas_sd8.jpeg]
